# Development of Functional Recovery Therapy for Post-Stroke Sequelae: Towards a Future without Stroke Aftereffects

**DOI:** 10.14789/ejmj.JMJ24-0026-P

**Published:** 2025-01-17

**Authors:** NOBUKAZU MIYAMOTO, NOBUTAKA HATTORI

**Affiliations:** 1 Department of Neurology, Juntendo University School of Medicine, Tokyo, Japan; 1 Department of Neurology, Juntendo University School of Medicine, Tokyo, Japan; 2 Neurodegenerative Disorders Collaborative Laboratory, RIKEN Center for Brain Science, Saitama, Japan; 2 Neurodegenerative Disorders Collaborative Laboratory, RIKEN Center for Brain Science, Saitama, Japan

**Keywords:** cerebral infarction, post-stroke sequelae, neurovascular unit, cell-cell interaction, mitochondrial treatment

## Abstract

Stroke remains a leading cause of mortality and morbidity globally, posing significant challenges to healthcare systems due to its impact on Activities of Daily Living, Quality of Life, and healthcare costs. Current treatments primarily focus on acute management through thrombolytic therapy and thrombectomy, but only a limited number of patients benefit, underscoring the need for effective therapies to aid chronic stroke recovery. Despite ongoing clinical trials, cell therapy faces substantial logistical and cost-related hurdles, limiting its widespread adoption. Strategies to minimalize post-stroke sequelae emphasize preventing cerebral infarction deterioration, utilizing predictive scoring systems for focused treatment, and exploring drug repositioning. The complex interplay within the Neurovascular Unit and Oligovascular Niche highlights the role of various cell types and neurotrophic factors in stroke pathophysiology and recovery phases. Notably, microglia and astrocytes exhibit dual phenotypes ─ either inflammatory or protective ─ depending on the environment, influencing neural damage or repair processes post-stroke. Mitochondrial therapy emerges as a promising avenue, leveraging the organelles' ability to migrate between cells and mitigate inflammatory responses. Studies suggest that mitochondria transferred from astrocytes or other sources could transform inflammatory astrocytes into protective ones, thereby promoting white matter integrity and potentially reducing dementia progression associated with stroke sequelae.

In conclusion, addressing stroke's multifaceted challenges requires innovative therapeutic approaches targeting inflammatory mechanisms and enhancing neuroprotection. Early detection and intervention, coupled with advancements in mitochondrial therapy and understanding intercellular interactions, hold promise for improving stroke outcomes and reducing long-term neurological complications.

## Introduction; stroke management: challenges and current status

If not properly prevented, strokes can recur, leading to a decline in Activities of Daily Living (ADL) and Quality of Life (QOL), eventually resulting in death. Stroke is currently the third leading cause of death, the fourth leading cause of morbidity, the leading cause of bedridden status (approximately 40%), accounts for 11% of elderly healthcare costs (12.1 trillion yen), and is responsible for one-third of cases requiring long-term care^1)^. It is also the primary reason for home nursing care utilization, with stroke-related sequelae, including dementia, imposing a significant burden on families and communities.

At present, the most notable recovery in stroke symptoms is achieved through hyperacute thrombolytic therapy and thrombectomy^2)^. Despite some expansion in treatment indications, only a limited number of patients benefit from these therapies. Beyond this, functional recovery is pursued through rehabilitation, making the establishment of effective therapies for chronic stroke recovery an urgent issue. In clinical practice, the limitation of treatment is the suppression of infarct expansion during the acute phase.

Although clinical trials for cell therapy are ongoing^3)^, the current status involves significant challenges. The preparation facilities required are substantial, and there are high costs associated with establishing closed-circuit systems and serum-free media for clinical use, ensuring product quality, and securing adequate quantities^4)^. Consequently, collaboration with drug companies is necessary, and these therapies have not to become widely available in clinical.

## Strategies to relieve post-stroke sequelae

Preventing the exacerbation of cerebral infarction is crucial for reducing post-stroke sequelae. To this purpose, we have developed a scoring system to predict patients at risk of deterioration, allowing for focused and intensive treatment^5)^. Additionally, we have created a deterioration prediction factor score specifically for penetrating artery territory infarctions, which are tend to worsening. In Japan, as the society moves towards super-aging, an increase in stroke patients is anticipated. Concerns have been raised about the disparity in physician distribution and the impact of stroke specialists on patient outcomes^6)^. To address these issues, we are developing a machine learning-based application for stroke diagnosis^7)^.

Furthermore, we aim to utilize drug repositioning, exploring the potential of clinically used medications such as ARBs^8)^, cilostazol^9)^, levetiracetam^10)^, pioglitazone^11)^, and GLP-1 agonists^12)^ to improve functional outcomes in stroke patients and slow the progression of vascular dementia. While these drugs have shown some efficacy in rodent models, their clinical effectiveness has been limited. This limitation is likely due to the inherent complexity of the brain's structure.

## The neurovascular unit and oligovascular niche

**Figure 1:**
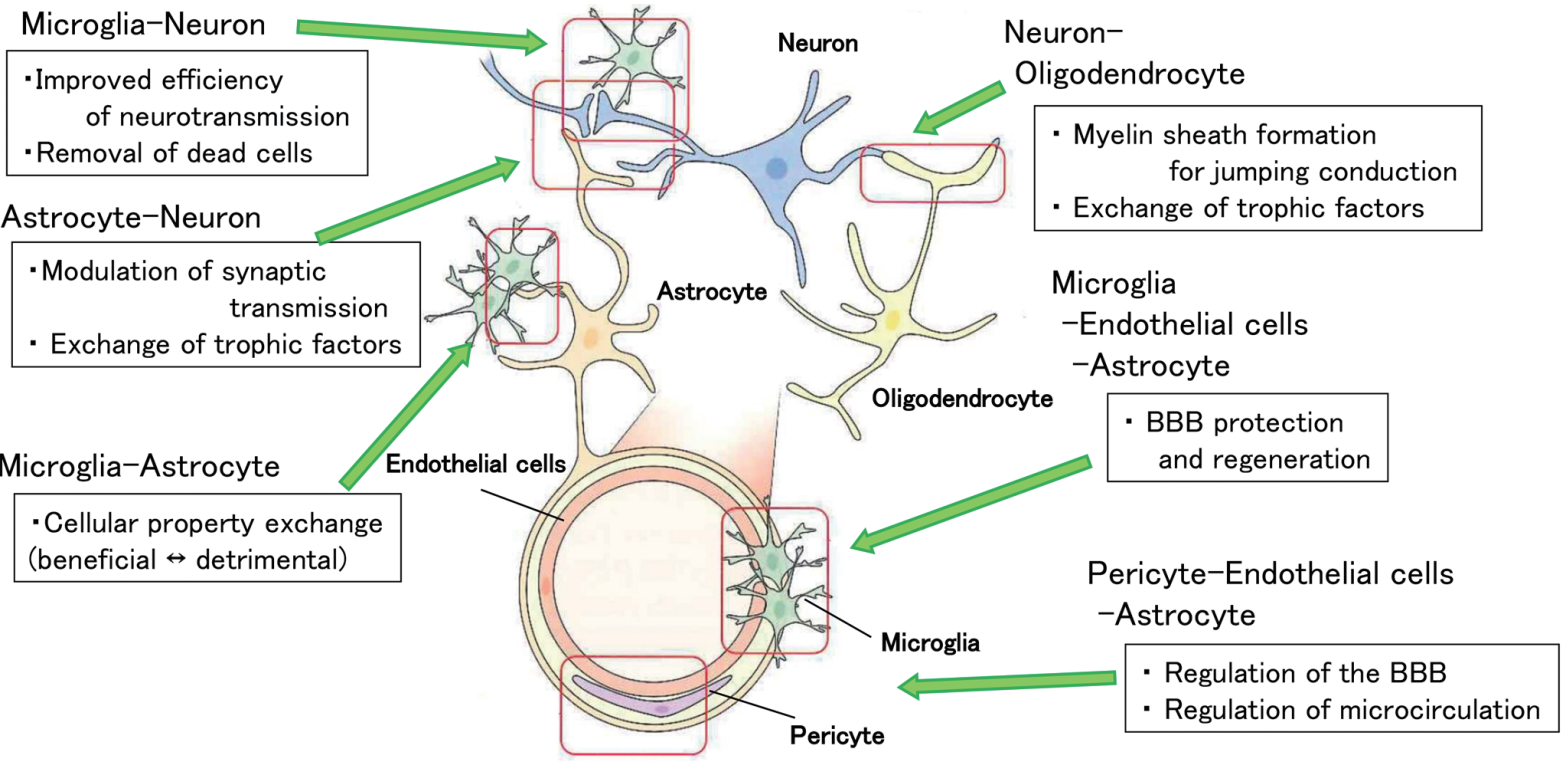
Component cells of the Neurovascular Unit and their role. Various trophic factors are exchanged between various cells.

**Figure 2:**
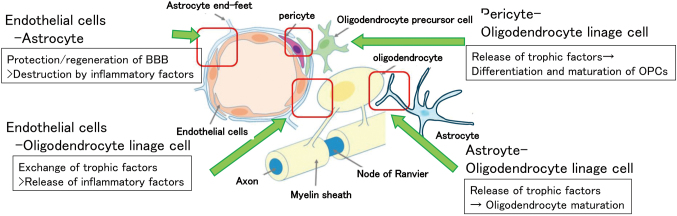
Component cells of the oligovascular niche and their role in the exchange of various trophic factors as in the Neurovascular unit.

The brain is anatomically divided into white matter and gray matter (cortex). The primary constituent cells of white matter include oligodendrocytes, astrocytes, and vascular cells (endothelial cells and pericytes). In gray matter, the main cellular components are neurons and astrocytes, supplemented by microglia and vascular cells (endothelial cells and pericytes). Recent studies have emphasized the significance of intercellular interactions. The Neurovascular Unit refers to the microenvironment of the neurovascular system in the cortex (Figure 1), while the Oligovascular Niche refers to the microenvironment of the neurovascular unit in white matter, emphasizing the interactions between oligodendrocytes and vascular cells (Figure 2). These interactions play various roles and functions, contributing to the maintenance of homeostasis.

Taking white matter as an example, oligodendrocytes are the main cells, and their continuous renewal is suggested to be crucial for maintaining white matter homeostasis. Under pathological conditions, the differentiation and maturation of these cells are impeded, leading to white matter degeneration^13)^. Astrocytes release various neurotrophic factors, including BDNF, PDGF, CNTF, and IGF-1. Microglia secrete IGF-1, TGF-β, and various other cytokines. Endothelial cells and pericytes release VEGF, bFGF, BDNF, and eNOS, contributing to the differentiation induction of oligodendrocytes and the migration of oligodendrocyte precursor cells^14)^.

Moreover, the dual-phase effects during the acute and chronic phases of stroke need to be considered. Inflammatory factors such as HMGB-1, NMDA, MMPs, ROS, and TNF-α are known to increase during the acute phase of stroke, leading to neuronal death and blood-brain barrier disruption. However, in the chronic phase, these factors play crucial roles in repair processes. Excessive inhibition of these factors might impede neurogenesis, angiogenesis, neural plasticity, remodeling, and the migration of oligodendrocyte precursor cells. Therefore, the key lies in managing the various neurotrophic factors, administering the right amount at the right time. Additionally, while the administration of a single neurotrophic factor may promote endothelial cell proliferation, it may be ineffective or even detrimental to oligodendrocyte precursor cells. This highlights the importance of the secretome, a collection of factors secreted by astrocytes and microglia^15)^.

## Impact of secretory cells

Is there a difference in the secretome depending on the secreting cells? Here, we present microglia and astrocytes as examples.

### 1. Microglia

Historically considered the cause of inflammation, recent studies have shown that microglia can adopt various phenotypes, such as inflammatory and neuroprotective, influenced by their environment^16)^. In conditions where inflammatory factors such as LPS, interferon-γ, and TNF-α are present, as seen in the acute phases of cerebral infarction, cerebral hemorrhage, or post-trauma, microglia become inflammatory. They release pro-inflammatory cytokines such as TNF-α, IL-1β, IL-6, and IL-12, leading to surrounding inflammation and tissue damage. Conversely, in the presence of cell debris or protective cytokines like IL-4, IL-10, and IL-13, microglia adopt a protective phenotype. They secrete anti-inflammatory cytokines such as TGF-β, IL-4, and IL-10, playing a role in anti-inflammatory responses and tissue recovery.

### 2. Astrocytes

Traditionally, astrocytes were considered to induce inflammation during the acute phase and promote tissue recovery during the chronic phase. Recent research has revealed that astrocytes, similar to microglia, can adopt either inflammatory or protective phenotypes^17)^. Under stimulation from cerebral infarction, cerebral hemorrhage, or trauma, astrocytes shift to an inflammatory state, releasing factors like interferon-γ and C1q, inducing neural damage and tissue injury. However, with anti-inflammatory factors or appropriate stimulation, astrocytes can release BNDF, VEGF, and bFGF, contributing to tissue recovery and the maintenance of homeostasis.

## Therapeutic strategy

**Figure 3:**
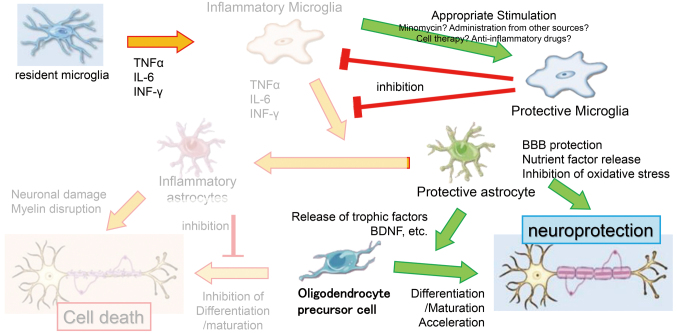
Therapeutic strategies based on various cell-cell interactions in stroke. The key is how to keep astrocytes and microglia with protective properties.

Based on the above, the ideal therapeutic strategy from the acute to chronic phases of stroke involves mitigating the inflammatory responses. Typically, after a stroke, inflammatory factors such as TNF-α and IL-6 transform resident microglia into inflammatory microglia, which in turn release similar inflammatory factors. This transformation changes the protective astrocytes into inflammatory ones, exacerbating neural damage, myelin sheath damage, and inhibiting the differentiation and maturation of oligodendrocyte precursor cells, leading to neuronal death and post-stroke sequelae. By administering appropriate stimuli, cell therapy, or anti-inflammatory drugs like minocycline, it is possible to inhibit the inflammatory transformation of resident microglia post-stroke. This suppression prevents the conversion of neuroprotective microglia to inflammatory microglia and inhibits the transition of astrocytes from a protective to an inflammatory state, thereby reducing the release of neurotoxic cytokines. Protective astrocytes contribute to maintaining the blood-brain barrier, releasing neurotrophic factors, and reducing oxidative stress, thereby promoting the differentiation and maturation of oligodendrocyte precursor cells and the extension of neuronal axons (Figure 3). This approach has the potential to alleviate post-stroke sequelae^18)^. Currently, in the field of basic research, it is not possible to clearly distinguish between inflammatory and protective microglia and astrocytes^19)^. Although very chaotic, cells with both properties have been found, and the results of future research are awaited. In addition, the successful utilization of these properties may be a trigger for posterior mitigation therapy.

## Pericytes

A crucial aspect of tissue regeneration is the restoration of vascular systems. Without adequate blood flow to the ischemic region, cells cannot receive the necessary nutrients, leading to cell death. Pericytes are mural cells embedded in the basement membrane and directly contact endothelial cells at the level of capillaries and small blood vessels. In the brain, pericytes are present in high ratios relative to endothelial cells, contributing to the formation of the blood-brain barrier and playing a crucial role in the regulation of cerebral blood flow. During tissue injury, some pericytes expressing PDGFRβ detach from the vascular wall and exhibit mesenchymal stem cell-like functions by secreting various trophic and immunomodulatory factors, thereby contributing to tissue repair and functional recovery^20)^.

## Intercellular interactions and the role of mitochondria

Recent studies have suggested the efficacy of exosomes in intercellular interactions^21)^. My focus, however, is on mitochondria. Mitochondria are intracellular organelles responsible for energy production in various cells, and their ability to transfer between cells has been reported. This phenomenon has shown potential benefits in myocardial infarction, pulmonary embolism, and dementia.

There is also evidence suggesting beneficial effects in stroke. Reports indicate that mitochondria extracted from astrocytes can migrate to damaged neurons, where they protect cellular functions and reduce post-stroke sequelae^22, 23)^. Similarly, mitochondria extracted from endothelial progenitor cells or preserved placental mitochondria have demonstrated comparable effects^24)^.

Our research has extended these findings to a vascular dementia model. We found that mitochondria released from astrocytes can transform inflammatory astrocytes into protective ones. This transformation promotes the division and maturation of oligodendrocyte lineage cells, which form the white matter, thereby inhibiting the progression of white matter lesions and dementia^25)^. This field holds significant promise for future research.

## Conclusion

Mitochondrial dysfunction has been implicated in the progression of stroke. Given the inevitability of post-stroke sequelae, early diagnosis of cerebral infarction and appropriate mitochondrial therapy could potentially prevent stroke onset and mitigate sequelae.

## Funding

This study was supported by JSPS KAKENHI (JP22K07355), and partly by: i) a grant from the High Technology Research Center; ii) a Grant-in-Aid for exploratory research from the Ministry of Education, Culture, Sports, Science and Technology, Japan; and iii) Grants-in-Aid from the Foundation of Strategic Research Projects in Private Universities from the Ministry of Education, Culture, Sports, Science, and Technology.

## Author contributions

NM conducted the experiments, analyzed the data, verified the results, and wrote the text. NH provided guidance and proofread the manuscript. All authors read and approved the final manuscript.

## Conflicts of interest statement

The Authors declare that there are no conflicts of interest.
